# Challenges and opportunities in embedding core components of substance use health care into Housing First programs

**DOI:** 10.3389/fpubh.2026.1817752

**Published:** 2026-08-26

**Authors:** Oluwaseyi Dolapo Somefun, Vicky Stergiopoulos, Sheena Taha, Nick Kerman, Leslie Buckley, Natalie Aubin, Alexander Caudarella, Stephanie Caroline Juhasz, Karly Muriel McCrone, Jodie Millward, Tomas Mirabelli, Kim Corace

**Affiliations:** 1 Centre for Addiction and Mental Health, Toronto, ON, Canada; 2 Department of Psychiatry, University of Toronto, Toronto, ON, Canada; 3 Canadian Centre on Substance Use and Addiction, Ottawa, ON, Canada; 4 Gattuso Centre for Social Medicine, University Health Network, Toronto, ON, Canada; 5 Centre de planification des services de santé en français/ French Language Health Planning Centre/Montfort Hospital/ Hôpital, Ottawa, ON, Canada; 6 RainCity Housing, Vancouver, BC, Canada; 7 The Neighborhood Group, Toronto, ON, Canada; 8 Department of Psychiatry, University of Ottawa, Ottawa, ON, Canada; 9 Ottawa Hospital Research Institute, Ottawa, ON, Canada

**Keywords:** Housing First, substance use disorders, homelessness, health policy, health services research

## Abstract

**Background:**

The intersecting crises of homelessness and substance use health (SUH) harms require a critical re-evaluation of mental health, SUH, and social care integration. Housing First (HF) is an evidence-based approach to ending chronic homelessness, but challenges in effectively supporting individuals with significant SUH needs within the intervention have not been well-documented. A national pan-Canadian workshop convened multidisciplinary partners to identify barriers, opportunities, and core components for the successful integration of SUH care within HF programs.

**Methods:**

The workshop involved a diverse group of attendees (*n* = 59), including direct service providers, people with lived and living experience (PWLLE), healthcare professionals, community leaders, researchers, and policymakers. Following brief presentations on key topics, a series of structured breakout sessions were conducted. Discussions were centred on two primary issues: (1) the key challenges and opportunities for integrating SUH care in HF programs, and (2) the core components and strategies required to advance the use of SUH best practices within HF.

**Results:**

Qualitative data collected from the breakout sessions revealed four key challenges: (1) fragmented services; (2) workforce challenges; (3) restrictive policy and funding environments; and (4) stigma. Opportunities for integration included: (1) strategic alignment; (2) cross-sector collaboration; (3) equity-informed use of data; and (4) flexible model adaptation. Core components for effective SUH integration in HF programs were identified as: (1) person-centred and flexible approaches; (2) foundational support for the workforce; and (3) integrated and collaborative care systems.

**Conclusion:**

Effectively integrating SUH care into HF requires a fundamental shift toward more person-centric systems: flexible, low barrier, culturally safe models that organize housing, health, harm reduction, treatment, recovery, peer, and community supports around individuals' self defined goals. The findings underscore the urgent need for coordinated policy action, grounded in evidence and lived expertise, to create a responsive system that upholds the right to both housing and health.

## Introduction

Homelessness encompasses a range of housing circumstances, including unsheltered homelessness (e.g., living on streets, in vehicles, or in parks), emergency shelter use, and temporary housing. People experiencing homelessness face profound health and social inequities compared to the general population (1). Mortality rates among unhoused individuals are higher than age matched peers, with markedly reduced life expectancy (2, 3). Chronic physical health conditions, including hypertension, diabetes, chronic obstructive pulmonary disease, and infectious diseases such as HIV, hepatitis C, and tuberculosis are also disproportionately prevalent in this population (4, 5). Mental illness affects an estimated 50%−80% of people experiencing homelessness, with high rates of post-traumatic stress disorder, major depression, and psychotic disorders (6). Traumatic brain injury is also overrepresented, often preceding or following experiences of homelessness (7). These health inequities are compounded by social exclusion, food insecurity, and barriers to accessing primary and preventive care, including stigma, lack of health insurance in some jurisdictions, and competing survival needs (8, 9).

The dual experiences of homelessness and harms from substance use represents one of the most pressing and complex public health challenges in Canada and other high-income countries (10, 11). The toxicity of the unregulated drug supply has driven mortality rates to unprecedented levels, while housing unaffordability and inadequate support systems led to increasing rates of homelessness (12, 13). Based on Canadian data from 2016–2025, opioid-related deaths peaked in 2023 (8,399) before declining modestly in 2024 (7,245), though long-term trends are still uncertain. The epidemic disproportionately affects men who account for approximately three-quarters of all deaths, with a mortality rate more than double that of women (14). Although mortality rates among adults aged 30–49 years remain the highest, the crisis is slowly shifting, with a declining share of deaths among 20–29-year-olds and a growing proportion among those aged 60 and older (14). While opioid related harms have received substantial attention, stimulant use (including cocaine, crack cocaine, and methamphetamine) is also prevalent among people experiencing homelessness, and is associated with increased morbidity, mortality, and mental health crises (15, 16). Further alcohol use disorder remains a leading contributor to morbidity and mortality in this population, with alcohol-related hospitalizations and liver disease occurring at rates many times higher than in the general population (17, 18). Notably, a bidirectional relationship exists between homelessness and substance use health concerns; each significantly increases the risk and severity of the other (19). The bidirectional link between homelessness and substance use is driven by interconnected mechanisms including trauma (such as childhood abuse or violence), which increases risks for both housing instability and substance use as a coping strategy (20); stigma toward people who use drugs, which restricts access to housing, employment, and healthcare (10); and untreated mental illness (like schizophrenia, bipolar disorder, or major depression), which creates a mutually reinforcing cycle of substance use and housing loss (21).

Housing First (HF) is an evidence-based intervention for ending chronic homelessness among individuals with mental illness (22). Its core principles, which include immediate, unconditional housing and person-centred supports, are typically delivered through Assertive Community Treatment (ACT) or Intensive Case Management (ICM) teams and have consistently improved housing stability and quality of life among participants (23, 24). However, recent reviews suggest that substance-use health (SUH) outcomes in HF have been equivocal, with little to no improvement in substance use health trajectories (25). As a result, individuals with unsupported SUH needs remain at elevated risk of overdose, health deterioration, recurrent instability, and preventable death even after rehousing (26–28). Recent evidence from permanent supportive housing (PSH) and HF programs reinforces these concerns, documenting rising overdose events and calls for augmentation of HF with more explicit addiction-specific care, treatment, and harm reduction interventions (29).

Existing literature has identified persistent barriers to integrating SUH care within Housing HF and other supportive housing settings, most notably systemic and policy-level challenges such as siloed funding structures, fragmented service systems, and punitive drug policies that criminalize substance use (30). While these factors do not directly contradict HF principles, they constrain implementation by limiting access to harm reduction, addiction medicine, and coordinated care within housing programs.

Despite increasing attention to the twin problems of homelessness and problematic substance use, critical knowledge gaps remain. First, most research has examined structural barriers to SUH-HF integration in isolation, leaving unclear how barriers interact across policy, funding, and service delivery domains to produce or compound implementation failures. Second, the relative priority of different solutions and which should be implemented first when resources are constrained has not been empirically established. Third, the perspectives of people with lived and living experience (PWLLE) are underrepresented in the published literature compared to those of providers or policymakers, despite strong evidence that PWLLE insights improve intervention acceptability and effectiveness (31, 32). Fourth, no national, multi-sectoral consultation has systematically synthesized expert consensus on the core components required to effectively embed a full spectrum of SUH care (spanning harm reduction, treatment, and recovery supports) within HF programs across Canada.

The present workshop was designed to address some of these gaps. By convening a national group of 59 experts including PWLLE, direct service providers, healthcare professionals, researchers, and policymakers, we sought to: (1) identify how systemic barriers to integrating SUH care into HF interact in practice; (2) generate priorities for action; (3) center PWLLE perspectives alongside other stakeholders; and (4) develop a consensus informed set of core components for integrating SUH care into HF. This paper reports the findings of that workshop.

## Methods

### Design and setting

A national, one-day round-table entitled “*Integrating Substance Use Health Considerations into Housing First Approaches*” was held on 26 May 2025. The roundtable was developed and delivered by the Canadian center for Substance use and Addiction (CCSA), and the center for Addiction and Mental Health (CAMH), and was conducted virtually using videoconferencing. Ten short presentations by experts (including PWLLE, service providers, researchers, and policymakers) focused on overcoming barriers and developing strategies to integrate SUH into HF programs. Seven breakout rooms were used to facilitate small-group discussions. Each room met twice to explore (1) the major challenges and opportunities for integrating SUH- care into HF, and (2) the core components and strategies necessary for integrating SUH best practices into HF programming. These sessions lasted approximately 40 min each.

### Attendees

A total of 95 individuals representing 61 unique organizations were invited by email to participate in the workshop. Attendees were identified using a purposive expert-consultation approach. The organizing committee invited individuals and organizations known through professional networks, prior collaborations, community and policy/practice meetings, and relevant publications related to HF, homelessness, SUH, harm reduction, healthcare, research, policymaking, and lived/living experience. The aim was not to recruit a statistically representative sample, but to convene a diverse group of stakeholders with direct expertise or experience relevant to integrating SUH supports within HF programs. Of the 95 individuals invited, 68 confirmed attendance, 16 declined, and 11 did not respond. Ultimately, 59 individuals attended the workshop, including nine presenters/committee members. A diversity of groups and perspectives was sought, including housing and HF providers, PWLLE, mental health service providers, SUH practitioners, primary care, public health, policymakers, peers, and community leaders. Each breakout room contained six to eight attendees, one facilitator, and one notetaker from the host organization.

### Participant characteristics

Workshop attendees represented six stakeholder groups. Table 1 presents the distribution of participants by role. People with lived and living experience (PWLLE) constituted 15% (*n* = 9) of attendees. Participants represented multiple jurisdictions: 20 (34%) from regional organizations, 12 (20%) from provincial organizations, and 27 (46%) from national organizations. Detailed demographic information (e.g., age, gender, ethnicity) was not collected to minimize data burden and protect anonymity in a small workshop setting.

**Table 1:** Attendee roles.

Attendee roles	Number (%)
People with lived and living experience (PWLLE)	9 (15)
Direct service providers (housing, outreach, case management)	24 (41)
Healthcare professionals (nurses, physicians, mental health clinicians)	15 (25)
Researchers and academics	14 (24)
Policymakers and government representatives	9 (15)
Indigenous representatives	5 (8)

Percentages are based on 59 unique participants. Some individuals hold multiple roles (e.g., a PWLLE who is also a service provider), so percentages sum to more than 100%.

### Data collection

Detailed notes were taken in real time by the CCSA staff members, with the convening steering committee acting as facilitators during each breakout session. The workshop was structured around three primary guiding questions posed to attendees in sequential sessions: (1) What are the top three challenges and opportunities for integrating SUH care into Housing First?; (2) What are the top three core components and strategies to integrate SUH best practices into Housing First?; and (3) Who else needs to be involved in implementing these changes? The notes captured attendees' responses to the guiding questions, follow-up- discussion points, and any consensus statements. Facilitators clarified statements when necessary and read back summaries to attendees for verification. Immediately after the round-table, the steering committee compiled the notes into a single document.

### Ethical considerations in workshop conduct

This manuscript reports on a knowledge-sharing workshop convened to identify barriers and opportunities for integrating SUH care into HF programs. The workshop was not designed as a research study a priori and therefore did not meet our institutions' definition of human subjects' research requiring formal research ethics board (REB) review. However, we adhered to core ethical principles of respect for persons, beneficence, and justice throughout.

#### Informed consent

At the start of the workshop, all attendees were informed that anonymized notes from breakout sessions would be used to generate a summary report and potentially a publication for knowledge dissemination. Continued participation in the workshop after this introduction conferred implied consent. Attendees could opt out of notetaking by notifying their facilitator; none did.

#### Equitable participation

Breakout rooms were limited to 6–8 participants to reduce power differentials and encourage speaking. Facilitators (CCSA steering committee members trained in facilitation) used a structured go-around at the beginning of each session to ensure every attendee, including PWLLE, had an opportunity to share their perspective before open discussion. Facilitators monitored group dynamics, invited quieter participants to speak, and gently managed dominant speakers.

#### Participant support

A staff member with mental health first aid training was available on-call throughout the workshop. Attendees were reminded at the outset that they could leave any session at any time without consequence. No distress incidents were reported.

#### Data security

No identifying information (names, organizations, geographic locations) was recorded in workshop notes. Notes were transcribed and stored on a password protected institutional server accessible only to designated members of the analytic team.

#### Compensation

PWLLE attendees received an honorarium of $30 per hour for their participation, consistent with our institutions' guidance on meaningful engagement of people with lived and living experience.

### Data analysis

Workshop data were analyzed using an inductive thematic analysis approach outlined by Braun and Clarke (33). The analysis followed a multi-step process. First, one author (O.D.S.) read the compiled workshop notes multiple times to become familiar with the data, noting recurring topics, areas of convergence, and areas of divergence across breakout groups. Second, O.D.S. conducted line-by-line open coding of the workshop notes using NVivo qualitative data analysis software. Coding was primarily inductive, with codes derived from the content of the notes rather than imposed from a pre-existing coding framework. However, the workshop's guiding questions on challenges, opportunities, and core components provided broad organizing domains that helped orient the analysis. Codes were short descriptive phrases capturing meaningful ideas or issues raised by attendees, such as “siloed services,” “staff burnout,” and “landlord stigma.”

Third, the initial codes were grouped into potential themes. For example, codes such as “siloed services,” “funding fragmentation,” and “lack of coordination” were grouped under the broader theme of “Fragmented Services.” Draft themes were then reviewed and discussed with two authors (S.T. and N.K.), who provided feedback on theme clarity, coherence, and fit with the workshop objectives. Where needed, themes were refined by returning to the workshop notes to ensure that they accurately reflected attendees' expressed perspectives. Fourth, candidate themes were reviewed against coded extracts and the full workshop notes to assess coherence, distinctness, and fit with the overall dataset. Overlapping themes were merged, and broad themes were separated where necessary. Fifth, the scope and focus of each theme were refined, theme definitions were developed, and representative quotes were selected to illustrate key findings. Finally, the Results section was written to present the themes coherently while ensuring that interpretations remained grounded in the workshop data.

Member checking was conducted with a subset of attendees (*n* = 8) to confirm the accuracy and resonance of the themes; their feedback informed final theme naming and interpretation. Throughout the analysis, we analyzed synthesized facilitator notes rather than verbatim transcripts, which we recognize as a limitation. To reduce the risk of misinterpretation, facilitators verified summaries with attendees during the workshop sessions, and notes were compiled immediately after the workshop. Presented quotes represent written notes of verbatim and paraphrased comments made by attendees during each breakout session.

### Researcher reflexivity

Following Braun and Clarke's (33) guidance on reflexive thematic analysis, we acknowledge the positionality and professional backgrounds of the authors involved in coding and interpretation. O.D.S. conducted the initial line-by-line coding of the workshop notes. S.T. and N.K. reviewed and discussed draft themes with O.D.S., contributing to theme refinement and interpretation.

O.D.S. is a health services researcher and demographer with expertise in mental health and homelessness research, whose analytic approach is informed by a primarily post positivist orientation. S.T. is a health services researcher specializing in harm reduction, substance use health, and workforce wellbeing, with prior frontline experience in community based substance use programs. Her analytic orientation is practice oriented, emphasizing actionable implications for service delivery and policy. N.K. is a clinical researcher with expertise in Housing First and supportive housing interventions. His approach is informed by a pragmatist perspective that recognizes both participant reported experiences and the broader structural conditions shaping them.

The remaining authors (V.S., L.B., N.A., A.C., S.C.J., K.M.M., J.M., T.M., K.C.) contributed to workshop design, facilitation, or manuscript review but did not participate in thematic coding. All authors reviewed and approved the final themes.

Consistent with the goals of the workshop, we adopted an experiential orientation to thematic analysis (33), prioritizing attendees expressed perspectives, meanings, and implementation experiences. This approach was selected because the primary objective of the workshop was to identify expert informed barriers, opportunities, and core components relevant to policy and practice discussions surrounding the integration of substance use health supports within Housing First programs.

### Results

Based on the objectives of the meeting, findings were categorized into challenges, opportunities, and core components needed to effectively integrate SUH care into HF programs (see Table 2).

**Table 2:** Summary of workshop findings on challenges, opportunities, and core components for integrating substance use health care into Housing First programs.

Challenges	Opportunities	Core components
Fragmented services	Strategic alignment	Person-centred and flexible approaches
Workforce challenges	Collaboration	Foundational support for the workforce
Policy challenges	Model adaptation	Integrated and collaborative care systems
Stigma		

### Challenges

Analysis of the breakout discussions revealed four key challenges, with their prevalence underscoring their systemic nature. Systemic fragmentation, the most frequently cited challenge (noted in all seven groups), was marked by silos between mental health, SUH, and primary care, creating discontinuous care pathways and treatment delays. Critical workforce challenges (a focus in six groups) including insufficient SUH expertise, staff wellness, and inadequate training stem from chronic underfunding and unsustainable caseloads. A restrictive policy and funding environment (raised in five groups) further impedes integration through siloed funding streams and punitive regulations. Finally, a pervasive culture of stigma from landlords and providers (also raised in five groups) reinforces these structural barriers, limiting housing access, and eroding person-centred care.

### Fragmented services

Attendees noted that fragmented services, and care discontinuity, created gaps during crucial transitions.

#### Siloed services

Attendees identified that a barrier to integration is the fragmentation across key systems including housing, healthcare, mental health, and income support, which operate with independent funding streams and competing priorities. The consequences of this structural divide were described as systemic silos operating at both policy and service-delivery levels. These silos fracture the system, making it a major logistical barrier to assemble essential healthcare supports. As one attendee emphasized, “*The degree of coordination is challenging… working with the province, city, feds for fundings, department of health, NGOs… when each department is meeting their own demands*.” This creates an overwhelming challenge for governance and seamless care delivery.

#### Continuity and comprehensiveness of care

Another theme that emerged was the systemic failure to ensure continuity and appropriateness of care, which manifested most acutely during transitions between levels of service and geographic locations. Attendees highlighted the lack of clear and reliable pathways for clients during critical transitions, such as from hospital to housing or from supportive housing to independent living. This absence of structured handoffs and poorly managed transitions was thought to cause clients to fall through the cracks and experience discontinuity in treatment. The human cost was captured by one attendee who described a “*disconnect between hospitalized and living precarious, discharged to a shelter but only at this point gets picked up from the housing team... People become destabilized again because of this gap*.” Another simply stated that clients were “*going here and there for different services*” without any coordination.

This challenge of continuity extended beyond transition points to a broader systemic fragmentation. Attendees noted that even when individuals were stably housed, the necessary SUH resources often weren't available in their community, forcing them to choose between maintaining their housing and accessing SUH support. As one attendee explained, “*The types of substance use resources aren't available where people are living, so they have to leave, and they might lose their place... Then we blame them because they are not engaging*.” While some organizations attempted to make care “*portable*” across municipal boundaries, primarily by informally continuing provider relationships or navigating transfers between regional systems, these efforts were described as inconsistent and at times ineffective. Participants noted that such approaches relied heavily on individual workers rather than formalized pathways, with one attendee explaining, “*We had someone transferred… but they were not able to take their supports*,” and another emphasizing that “*there's no systematic way for people to be connected to health care*.”

Concerns were especially acute for aging populations, as attendees worried that “*there are not that many great transitional supports… Will there be adequate care all around?”* These shortcomings left some programs facing the reality of supporting people at the end of their lives without the necessary infrastructure: “*We always have people who can't go into senior facilities because of their substance use, but they are being evicted because of it. We take them, but we are not equipped to help them with their medical needs. We have people dying in the programs—we do not do end-of-life care, we are not equipped for this.”* Collectively, these accounts underscore that supporting continuity and comprehensiveness of care remains a fragile and uneven aspect of HF programs, with significant implications for stability, engagement, and long-term health outcomes.

Attendees further emphasized that challenges to continuity and comprehensiveness of care were compounded by geographic barriers, particularly in rural, remote, and Indigenous communities. Participants noted that essential substance use health services are often not co-located with housing, requiring individuals to travel long distances to access care—an expectation that was described as unrealistic for many residents, particularly those without transportation. As one attendee summarized, there is a “*lack of colocalization of services/supports with housing*,” while another explained that “*the types of substance use resources aren't available where people are living, so they have to leave, and they might lose their place*.” These geographic constraints were seen as exacerbating service disengagement and housing instability, especially in remote contexts where “*people who aren't near enough to some of these resources… whether they exist or not, you're not close enough to be able to act*.”

### Workforce challenges

Frontline staff and organizations face critical barriers that hinder their ability to effectively integrate SUH care, primarily stemming from insufficient support. These challenges were categorized into three sub-themes:

#### Staff wellness

Frontline workers described experiencing distress and moral injury primarily as a result of systemic and organizational conditions, rather than from their work with clients. Participants emphasized that high-stress environments, overwhelming administrative and coordination demands, and the need to navigate fragmented and restrictive policy landscapes create sustained strain on staff, compounded by a lack of dedicated mental health supports for the workforce itself. Attendees described feeling overwhelmed by wanting to serve their client's and the emotional toll of witnessing trauma and death. One noted: “*Staff burnout and moral injury- frontline peer support network. Doctors have one, would be nice if provincially or nationally if there was some sort of peer support line*.” This was iterated by another staff: “*6 months working in this without burnout... Capacity much lower after 6 months, need to put in strategies to support folks, if you don't you're churning in and churning out.”* Another attendee explicitly put it this way: “*What I see as burnout, not from the work with people, it's from the systems that we work in. People know how to meet their own needs. It's all the factors, there is no movement, toxicity from the systems you work in. This is the burn out question. This is the piece. Those of us who have worked in this, we aren't here to fix anything, when you must fight across systems, feels like you are putting your head through a wall.”*

#### Large caseloads

Attendees described how heavy caseloads and low wages for the workforce stem from inadequate funding. As one provider explained, “*Being short staffed is always an issue*,” noting that staff must manage more clients than is sustainable. Attendees stressed that without stable funding it is difficult to recruit and retain staff, leading to chronic understaffing and high turnover rates, impacting quality of care. For instance, “*A lot of staff have to work two jobs to survive... they end up going [to hospitals] because they can't afford living [on community salaries]*.” Another added: “*Frontline workers are underfunded- they're accessing poverty reduction supports while trying to support people in crisis.”*

#### Gaps in expertise and specialized training

Another critical impediment to effective care was a systemically under-developed workforce, where direct service staff were expected to manage individuals with intersecting needs without adequate, specialized training. A fundamental competency gap existed, as housing staff often lacked training in SUH, whereas healthcare providers frequently operated without an adequate understanding of Housing First principles. This disconnect was seen to lead to inconsistent and potentially harmful client interactions, a risk captured by an attendee who questioned the system's preparedness: “*SUH is a distinct domain, housing first, see folks being supportive but not specialized in this field... Are people actually properly trained and supported to understand the nature of the needs they are hoping to support*?”

This lack of expertise was not attributed to a lack of staff interest, but rather to a systemic failure in professional development and technical support. Attendees highlighted that “*HR challenges are very real. Even those who are interested in the training can't get the support,”* and noted a specific “*lack of training and technical support*.” The consequence was that a well-intentioned workforce was often set up to fail. In response, there was a strong desire expressed for more robust learning structures to bridge these gaps, with one attendee observing that “*People want a community of practice even, to hear from other Housing First programs*.” This highlighted a clear need for accessible training in harm reduction, best practices in concurrent health and trauma-informed care, as well as peer learning networks to build a truly integrated, competent workforce.

### Policy challenges

Attendees identified a challenging policy environment as a major barrier to integrating SUH care within HF programs. Across breakout discussions, three interrelated policy sub-themes emerged: (1) criminalization and punitive drug policy; (2) a policy retreat from harm reduction; and (3) housing market governance and landlord gatekeeping. Together, these dynamics were seen to constrain the ability of HF programs to deliver a comprehensive, person-centred spectrum of SUH care spanning harm reduction, treatment, and recovery supports despite broad consensus on what works.

#### Punitive drug policy environments

Attendees across multiple breakout rooms described an increasingly punitive policy climate that criminalizes substance use and undermines the integration of SUH care within HF. Attendees cited recent policy developments in Ontario, including Bill 6, which criminalizes public drug use, and Bill 10, which allows housing providers to refuse tenancy based on a prospective tenant's substance use. One participant noted that it “*feels there is a political crack down on people who use substances*,” while another cautioned that “*as we move more and more to criminalization of people who use substances, this is one of the barriers we might see coming up*.” These policies were viewed as fundamentally misaligned with the low-barrier ethos of HF, creating fear, instability, and exclusion for people most in need of support. One attendee further emphasized that criminalization reshapes public discourse, positioning substance use as a public safety issue rather than a health concern. They explained: “*Messaging blames the people who use substances because they are unhoused; social disorder on the street, people who use substances are being blamed, when really the issue is homelessness and lack of housing*.”

#### Policy retreat from harm reduction

In addition to criminalization, attendees identified a policy environment that retreats from evidence-based SUH care, with harm reduction being a prominent example. Participants described a growing shift in policy and practice away from harm reduction approaches, characterizing this movement as “counterproductive” and symptomatic of a broader failure to fund and support a full continuum of SUH services. Several attendees emphasized that harm reduction is increasingly positioned as incompatible with recovery or public safety, despite strong evidence to the contrary, and that this false dichotomy neglects the need for accessible treatment and addiction medicine. For instance, one attendee said: “*The discouragement with harm reduction is a false dichotomy. It's gained traction politically but isn't evidence based*.”

Attendees expressed concern that this retreat reduces options for people who are not seeking abstinence-based outcomes and undermines efforts to meet individuals where they are at in their substance use and recovery trajectories. For instance, this attended noted: “*If we are going to be looking at things from an honest perspective, let's look at different goals that we are allowing people to have*.” Several noted that harm reduction is being politically reframed as incompatible with recovery, rather than as a foundational component of health and safety. This narrowing of the continuum of acceptable interventions was seen to undermine HF's capacity to offer person-centred care, whether that involves harm reduction, access to treatment, or support for recovery goals.

#### Housing market governance and landlord gatekeeping

Finally, attendees highlighted housing market governance, and particularly the role of private landlords as a key policy-related barrier. Participants described landlords as essential gatekeepers within a housing system where units are treated primarily as financial assets. Several noted that policy uptake of HF recommendations is limited because landlords “*want to protect their investment and get nervous*,” particularly when HF programs are negatively portrayed in the media. Another added: “*Landlords know what the program is and they have these ideas about the clientele and will outright ask questions about them, on their use, so you can tell that landlords are trying to protect themselves right off the bat, so we try to converse with them and break down barriers*.”

Attendees further noted that these dynamics are shaped not only by stigma, but also by housing form and building configuration. Concerns were raised about the concentration of Housing First tenants within specific buildings, particularly where HF residents constitute the majority of tenants, which was described as intensifying landlord reluctance and community scrutiny. As one participant observed, “*as we stand now, most of HF individuals are housed as the majority in certain buildings*,” a configuration that was seen as heightening visibility and reinforcing negative perceptions. At the same time, dispersed and single-unit housing models were described as presenting different challenges, including social isolation and increased substance-related risk. One attendee noted that when people are housed alone, “*they become isolated, and their only connections are professional — you lose community*,” underscoring how building form can limit opportunities for social connection and the feasibility of co-locating harm reduction or substance use health supports within residential settings.

This dynamic was compounded by insufficient policy mechanisms to mitigate landlord risk, such as damage funds or dedicated housing supports, leaving HF providers to negotiate access to housing on a case-by-case basis. Attendees also emphasized that stigma toward people who use substances influences landlord behavior and housing availability, reinforcing exclusion despite HF's evidence base. As one participant noted, negative public and media narratives about HF discourage landlord participation and further constrain housing supply for people with complex SUH needs.

### Stigma

Stigma emerged as another challenge to SUH care within HF programs. It operates at systemic, societal, and interpersonal levels, directly shaping housing access and the policy environment. As one attendee noted, “*the use of substances is a quick and easy way to exclude someone from housing*,” a practice evident in non-HF settings where “*the bar is getting higher*” for those seeking housing. This exclusion is particularly evident for older adults with SUH needs as noted by an attendee: “*SU is a check box—they are automatically not even considered*.”

These exclusionary practices are rooted in broader systemic stigma. Participants observed that “*policies are contrary to what we need to do*,” and that “*the political environment and public stigma*” constrain available housing and support options. Media narratives reinforce this by framing substance use as a public disorder issue rather than a health issue rooted in multiple social and systemic factors including homelessness. As one attendee explained, “*the messaging blames the people who use substances because they are unhoused… when really the issue is homelessness and lack of housing*,” adding that “*people prefer to be housed, regardless of their [substance use], and then they wouldn't be causing public disorder if they had a place to use in private*.”

This stigmatizing environment surfaces directly in daily operations, affecting interactions with landlords and service providers. One attendee asked, “*Is there a program or how does Housing First educate and destigmatize the issues with the landlords?”* Negative media portrayals were seen as a key barrier, fueling doubt about landlord alignment with program goals as noted by this attendee: “*challenges between landlords and Housing first. HF has received negative portrayals in the media. Is HF more effective when it's outside of the private realm? Do the goals of the landlords align with those of HF*?”

Ultimately, this environment not only restricts housing availability but also degrades service quality, as staff attitudes and institutional bias create judgmental care environments that impede the expansion of SUH supports within Housing First.

### Opportunities

There were three interrelated themes related to opportunities to integrate SUH considerations into HF programs: strategic alignment, collaboration and leveraging the use of data, and model adaptations. Strategic alignment and collaboration were raised across all breakout groups, while the importance of data use and model adaptations was discussed in most groups, though with varying emphasis.

### Strategic alignment

#### The need for a collective, unified voice

The need for strategic alignment emerged as a critical prerequisite for overcoming policy challenges and stigma. Attendees emphasized that progress required a cohesive, multi-sectoral strategy that brought together organizations, sectors and levels of government, moving beyond isolated efforts. Discussions like these represented a vital step toward building that unified front, yet participants stressed the need to translate dialogue into actionable policy guidance. One attendee captured this call to action: “*I would love to see housing first providers to develop a white paper around what we are missing, what can we do in this environment? A collective voice around this. Governments will do what they will do, and that can change quickly, but there is room to have more of a collective voice around this*.” This was supported by another who stressed the foundational step of “*Getting governments on board. Educating governments*.”

Attendees articulated that this alignment must be multi-layered, strategically engaging actors across public and private sectors to create a powerful coalition. The counterproductive nature of higher-level policies was noted, underscoring the need to align efforts across all levels of government. As one attendee advised, “*We need to target municipalities, where they do have control, you can move more nimbly. If you can get a strong political champion like a mayor. We don't talk about the private sector enough… get prominent business leaders that it's good for the economy including these people will have more political clout*.”

Attendees identified potential champions such as mayors, business leaders, and unions, the latter of which “*can advocate… through collective action*” for wage parity and better working conditions.

#### Centring lived experience

There was a strong consensus on the critical need to integrate the powerful stories and expertise of People with Lived and Living Experience (PWLLE) alongside evidence and research to address knowledge gaps and enhance the person-centred implementation of HF. Peer-run organizations and PWLLE experts were viewed as indispensable, not only for shaping program designs to reflect real needs but also for holding systems accountable to harm reduction and HF principles. As one attendee emphasized: “*Peer-run organizations can shape program designs to reflect real needs and hold systems accountable for harm reduction and housing first principles.”* This direct testimony was seen as crucial for both effective messaging and accountability, as detailed by an attendee: “*one individual shared how explaining their experience to communities helped the people in that community understand the importance and value of certain supports.”*

The discussion then turned to how to ensure these narratives are heard meaningfully. One proposed method was a “*partnership or visual representation... a personal experience map. So what it is like for someone go through all these different stages from their perspective, and I think that kind of humanizes things*.” This approach aligns with the observation that “*one of the useful outputs is to have visual representation of all those stories that can become physical and used as evidence*.”

Finally, one attendee acknowledged that this work requires careful support, recognizing that “*we need PLLE in the room AND it takes a different finessing and supporting, especially if its fresh PLLE. They need a unique set of skills to support them*.” This ensures that the process is not only effective but also ethical and empowering for those sharing their experiences.

#### Advocating for specific policy changes

The discussions revealed that advocacy must be directed toward clear, actionable policy goals across the full spectrum of care. Attendees emphasized that advocacy should not be vague; it must focus on concrete policy shifts and ensure that no element of evidence-based care is removed from service provision. This included strong calls for better funding and timely access to mental health and substance use health care, as noted in the facilitator's summary: “*Too long to receive SUH supports (e.g., treatment) when in HF. Waiting months, people can fall back or have difficulty staying committed*.” Another participant pointed to the need for integrated care models, stating, “*The siloing between MH and addictions treatment, try to bring them together*.” Alongside these systemic calls, harm reduction remained a critical advocacy pillar, with one attendee urging: “*Advocating for decriminalization, changing the discourse on public safety*,” and another stressing the need to “*advocate for safe use settings in Ontario, and safer supplies like needles*.” These points collectively underscored that effective advocacy must address the whole spectrum of needs ensuring HF models are supported by comprehensive, well-resourced, and collaborative care systems.

### Collaboration

#### Interdisciplinary collaboration

Attendees stressed that housing, healthcare, addiction services, and mental health are currently siloed. They highlighted that success depends on creating integrated, multidisciplinary teams that can wrap supports around the individual, breaking down silos between housing, healthcare, and social services to provide holistic, person-centered care. One attendee mentioned: “*Building strong cross sector partnerships. This could lead to coordinated care*.” This was echoed by another attendee: “*Embedding a multidisciplinary mobile support team, having a substance use counsellor, mental health counsellor etc all under one roof* ” and “*Municipalities play an increasing role, without their support the work we had in place would have never gotten off the ground. This partnership is critical along with community organizations and hospitals.”*

The importance of strategic champions and partnerships was also noted. For instance, one attendee who directly works in a housing program highlighted the growing role of public health authorities as key allies, noting: “*Our local public health unit is more and more recognizing that housing is a large determinant to health, so they have become an advocate…*” This reflects a broader trend of health-sector actors aligning housing stability with public health outcomes.

#### Partnership with landlords

Many of the attendees agreed that a successful HF model requires collaboration beyond the health and social sector. Engaging and educating private landlords was presented as a critical, though challenging, component. The suggested approach was to actively recruit, educate, and support private landlords to overcome stigma and secure housing stock. For example, one attendee mentioned: “*building a repour with the landlords with HF staff, so that landlords feel supported by HF staff regarding damages, etc.”*

#### Using evidence for advocacy

Attendees identified a clear need to use concrete evidence and data to educate decision-makers and shift political and public narratives that are often not based on facts. This would require translating the existing, strong evidence base into accessible forms for advocacy. As one attendee noted: “*This conversation needs to get out to decision makers; may have heard some of the language but don't understand.”*

Leveraging this growing evidence base was viewed as a critical way to advance substance use health within HF programs. Participants stressed the importance of grounding programs in proven pathways, with one stating: “*Housing First should remain with its original pathway—evidence supports that.”* Specific examples of promising programs were offered to illustrate this point. One attendee shared: “*Study at a [health centre]: program still exists today, was housing and supporting people, who had severe addictions. It was matched sample, not RCT. Difference showed up where people did better around alcohol consumption and related issues who were receiving treatment.”* Another added from their own context: “*[xxx] has piloted study, they have to have opioid addiction and have to be unhoused, they have gotten people housed, it's promising.”* These examples underscored that using local, concrete data is key to demonstrating effectiveness and securing support for integrated models.

#### Need for more data

Others called for explicit research and data collection to counter skepticism and guide scaling: One attendee suggested: “*add a research element because we aren't hearing about the good work that's being done – can be a form of advocacy and knowledge mobilization*,” and another attendee pointed out the “*need for more data*, stating that: “*New environment and new landscape call for data in real time. This may be a missing piece for the SU population*.” In response, one attendee emphasized staying “*within what we know has shown to work*” and incorporating substance use health “*in an evidence-based way*,” while another attendee noted that “*it is nice to provide metrics rather than no metrics*.”

### Model adaptation

#### Flexible models

Another theme emerging from the discussions was the critical need for service models that extend beyond traditional approaches to offer more flexible and person-centred support. As one attendee noted, “*One thing we are working on internally is to make their care as portable as possible, as they move, we are still able to provide the medical care they are receiving in one area to another*,” highlighting the necessity of robust mobile support to maintain care continuity. This need for mobility was directly linked to the geographical challenges clients face, as another attendee observed that “*people are having to travel a fair distance to get to the resources*,” suggesting that “*looking at mobile resources*” and “*having spaces available and approved*” in more locations was a crucial consideration. To address this, one attendee suggested: “*having numerous buildings where an individual could be moved if one spot wasn't working out; but not relocated a far distance; just within the “complex” of multiple buildings that could work out instead of having the person leave [the local area] entirely.”* Also, there was a suggestion to blend HF units with general housing by one of the attendees: “*the Finnish model uses public and private units. People will often choose to be in a congregate housing setting and then eventually choose to be living on their own. [There is talk about] private entrepreneurs purchasing large groups of modular housing and making a profit by supplying affordable housing privately*.”

#### Multidisciplinary teams

Another suggestion was moving away from siloed services to fully integrated teams that bring care to where people live. One attendee said: “*Embedding a multidisciplinary mobile support team, having a substance use counsellor, mental health counsellor etc all under one roof this could really benefit the tenants... because many don't like traveling to doctor's appointment.”* Another attendee added: “*one of the components of the HF model has an integrated team... You should basically have ACT teams. Some of the navigation is critical in the beginning.”*

### Core components for integrating SUH within Housing First programs

Building on the identified challenges and opportunities, participants identified three interrelated core components essential for the effective integration of SUH care into HF programs. Each component directly responds to specific gaps discussed earlier, offering actionable pathways forward without reiterating the full context of each challenge. The section presents three interrelated themes: (1) Person-centered and Flexible Approaches, (2) Foundational Support for the Workforce, and (3) Integrated and Collaborative Care Systems.

### Person-centred and flexible approaches

This theme emerged in response to gaps in rigid program structures, geographic barriers to care, and misalignment between client goals and service expectations. Attendees consistently emphasized the need for HF to remain flexible and centred on individuals' self-defined goals rather than imposing rigid program criteria. While some tenants seek respite or stability rather than abstinence-based outcomes, others actively pursue treatment or abstinence. The core principle is responsiveness to personal choice, whether that means supporting harm reduction, facilitating access to recovery-oriented housing, or enabling moves to environments that align with an individual's chosen path. As one attendee noted, “*The individuals were not looking for treatment, they were looking for respite… If we are going to be looking at things from an honest perspective, let's look at different goals that we are allowing people to have*.”

Participants further emphasized that person-centred care requires not only flexibility at the point of housing entry, but also mobility after housing placement. Attendees described how individuals' substance use health, recovery, and care needs often change over time, yet once housed, it can be difficult to move to settings better aligned with evolving goals, access services, or reduce isolation without risking housing loss. As one attendee observed, “*The types of substance use resources aren't available where people are living, so they have to leave, and they might lose their place*,” highlighting how limited mobility can force people to choose between housing stability and care.

Client agency and choice were highlighted ensuring clients have real choice in their housing type, service engagement, and life goals to foster environments conducive to recovery. This includes involving them in designing their own environment and rules that support their SUH goals (e.g., resident-led harm reduction agreements). As put by one of the attendees, “*It's about working with the individuals and giving them the chance to control the type of environment they recover in (resident leadership vs resident involvement)*.”

### Foundational support for the workforce

This theme was responding to gaps in staff burnout, inadequate training, high caseloads, and high turnover. Another prominent theme was the critical need for foundational workforce support, addressing the distress and lack of resources experienced by frontline implementation staff. Key recommendations centred on comprehensive training, competitive compensation, and mental health support.

A primary concern was equipping staff with the necessary skills to manage the complexities of the current substance use crisis. As one attendee noted: “*Staff feel unequipped to handle the substance use disorder they see today… need long-term strategic planning and more tools.”* To bridge this gap, participants called for mandatory, ongoing education in overdose prevention, trauma-informed care, and de-stigmatization. This need was iterated by another attendee*: “equip the HF program with overdose prevention strategies... Training should be offered; we currently do not offer enough to our HF program.”*

Beyond individual training, there was a strong push for systemic knowledge-sharing through a “*national community of practice,”* where practitioners could learn collaboratively. This was seen as essential for sustainability and scaling effective models, moving beyond isolated efforts.

However, training alone was deemed insufficient without addressing job quality and wellbeing. Stakeholders emphasized that low wages drive high turnover, as highlighted by this observation: “*Large pay gap, staff get lost to provincial and municipal services because they can earn more money.”* Competitive compensation is crucial to retain qualified professionals.

Finally, protecting staff mental health was a major priority to prevent burnout and moral injury. This requires creating safe, accessible support systems. One attendee pointed out a key barrier: “*Having a place where frontline workers can get psychological support to prevent burnout… but many are afraid their employer will find out.”* This underscores the need for confidential, robust supports, including peer support lines and protected debriefing time. The overall strain was summarized concisely: “*Burnout and workload on front line staff… staff need self-care training, and management needs training as well.”*

### Integrated and collaborative care systems

This theme was responding to gaps in service silos, fragmented funding, poor cross-sector coordination, and landlord reluctance. Attendees suggested the dismantling of silos to integrate housing, health, and social services. First, the co-location of interdisciplinary teams was suggested as a primary method, involving the embedding of a multidisciplinary team within housing settings. As one attendee suggested: “*How do we have ACT teams, or interdisciplinary teams, who work in tandem with the housing staff?”* Another reflected on successful models, noting*, “In some sites we had an ACT intervention — housing was available, and once you situate supports there…food security, medication, health care — it works when the team becomes the pathway.”* The development of strong cross-sector partnerships was also identified as crucial, necessitating formalized agreements with health authorities and community agencies to facilitate care coordination.

Furthermore, experts emphasized the need for defined pathways to care, specifically the creation of streamlined, low-barrier referral processes to connect clients directly to healthcare and mitigate institutional discharge into homelessness. As noted by this attendee, “*There's no systematic way for people to be connected to health care — it depends on the individual worker. We need designated pathways to care*.”

## Discussion

This national multi-stakeholder expert workshop successfully identified the key challenges, opportunities, and core components essential for integrating SUH care within HF programs across Canada. Our findings delineated critical systemic barriers including fragmented services, a pervasive culture of stigma, and serious challenges in workforce capacity that currently hinder this integration. This section interprets these findings in the context of existing literature and highlights their implications for policy, practice, and future research.

HF is built on the principle that housing is a human right and should be provided without treatment prerequisites (34). However, findings from our workshop highlighted that many HF models lack embedded SUH services (28). This critique is consistent with evolving research on integrated care models. While early critiques noted limited implementation of harm reduction within HF (35), the field has since progressed, with harm reduction increasingly recognized as a foundational component of many HF programs. However, this shift has not been matched by commensurate development of other essential supports, such as addiction medicine, evidence-based treatment, and intensive mental health services (36). To fully realize HF's promise as an intervention for chronic homelessness, a comprehensive and integrated spectrum of SUH care, spanning harm reduction, treatment, and recovery must be systematically embedded within housing programs. Motivational interviewing and integrated treatment for concurrent disorders should be core, funded components of these models, rather than adjunct or inconsistently available services.

Although our focus was on augmenting HF interventions to better support people experiencing homelessness and SUH needs, alternative and complementary housing models may also have a role within a broader continuum of community supports. For example, Oxford Houses are democratically run, self-funded, peer supported recovery homes that require abstinence as a condition of residency and have shown promising outcomes in substance use recovery literature, particularly in U.S. based studies (37, 38). However, because Oxford Houses are abstinence contingent, they may not be appropriate for all individuals, particularly those with ongoing substance use, relapse risk, or a preference for harm reduction-oriented supports. Similarly, single site permanent supportive housing with 24/7 onsite support may better meet the needs of individuals requiring more intensive and continuous care. Rather than positioning these models as replacements for HF, a diverse housing ecosystem that includes low barrier HF, recovery-oriented housing, and higher support permanent supportive housing may better respond to the heterogeneous needs and goals of people experiencing homelessness and SUH concerns.

Attendees identified punitive drug policies as a key obstacle to integrating SUH care into HF programs, creating a direct conflict with the low-barrier, harm-reduction inclusive approach that is central to Housing First. Evidence from the overdose literature underscores the consequences of this misalignment, revealing that most overdose deaths occur inside housing environments because people use drugs alone, and cannot access supervised consumption services or safer supplies (39). In British Columbia and some cities across the United States, a disproportionate number of fatal overdoses occur within housing settings (40), and the rate in single-room occupancy (SRO) facilities is as much as 20 times greater than that of the general population (41). However, our findings indicate that although embedded harm reduction services are an important element for mitigating overdose risk, they are not sufficient on their own. A comprehensive public health approach must be grounded in additional core components identified through the workshop discussions. First, models must be person-centred and flexible, respecting client autonomy, self-defined goals, and pathways that do not hinge on abstinence. In this context, person-centred systems are flexible, low-barrier, and responsive to individuals' changing needs and self-defined goals, rather than requiring people to fit into rigid, siloed, or abstinence-oriented service structures. This includes real choice in housing and supports, access to harm reduction, treatment, and recovery supports, mobility across housing options, and coordinated pathways across housing, health, and social care. Importantly, workshop discussions emphasized that such flexibility must extend beyond initial housing placement to include mobility within and across housing options, as static placements may become misaligned with individuals' evolving substance use health, recovery, aging, or care needs. Second, sustainable implementation requires foundational support for the workforce, including specialized training in substance use health, mental health and trauma-informed care, competitive compensation, and access to mental health resources to address burnout and moral injury. Third, truly integrated and collaborative care systems are needed to dismantle entrenched silos between housing, health, and social services, ensuring timely access to substance use health care and treatment through seamless pathways rather than fragmented service delivery.

Although the challenges identified in this workshop were described as system wide barriers, they are unlikely to be experienced evenly across populations. For Indigenous, Black, racialized, and other minoritized people experiencing homelessness and substance use harms, fragmented services, punitive drug policies, stigma, and limited access to housing are shaped by broader histories and contemporary forms of structural racism and colonization (42). In the Canadian context, these include ongoing inequities in housing, healthcare, child welfare, policing, and criminal justice systems, as well as limited access to culturally safe and community led substance use health supports. As a result, policy and service barriers may compound existing forms of exclusion and mistrust, particularly when care systems fail to address the social, cultural, and historical contexts that shape people's experiences of homelessness and substance use. Integrating SUH care into HF programs therefore requires not only better coordination across housing and health systems, but also explicit attention to anti-racism, decolonization, cultural safety, and Indigenous and community led models of care.

Our findings emphasized the need to address fragmented services and siloed funding. Research on the homeless service sector shows that “funding silos,” separate streams for housing, physical health, mental health and substance use health services create disconnected care systems (43–45). Our findings extend this understanding by highlighting the consequences. This fragmentation does not merely create administrative inefficiency; it actively prevents HF programs from providing the very core components deemed essential for success. In contrast, a person-centred system would organize funding, services, and care pathways around the individual's needs and goals, rather than requiring individuals to navigate separate housing, health, mental health, and SUH systems on their own. Blending funding across housing and health ministries is therefore not just a fiscal reform, it is a prerequisite for operationalizing the integrated care model attendees described. Such a shift is necessary to co-locate interdisciplinary teams, ensure reliable access to primary and SUH care, and move beyond a sole focus on harm reduction to provide a comprehensive spectrum of care that includes prevention, treatment, and recovery supports. Participants' concerns also reflected a tension between episodic, crisis-driven medical care and the need for longitudinal, relationship-based primary care within Housing First, particularly given the long-term nature of housing placements. Reliance on intermittent or emergency-based care was viewed as misaligned with ongoing substance use health and complex medical needs.

Equity oriented and anti-racist approaches are also essential to advancing the opportunities identified in this workshop. Strategic alignment, advocacy, collaboration, data use, and model adaptation should explicitly address how structural racism, colonization, criminalization, and housing exclusion shape access to housing and SUH care. This includes centring Indigenous, Black, racialized, 2SLGBTQ+, immigrant, and other minoritized voices in leadership and decision-making; partnering with Indigenous- and community-led organizations; using equity-informed data carefully; and prioritizing culturally safe, trauma-informed, and community-led models of care.

A recent scoping review reinforces attendee reports of a staffing crisis, linking the high distress and turnover among homelessness workers to intense job demands, limited resources, and consequent emotional strain and secondary trauma (46, 47). Attendees suggested specialized training in trauma-informed care and overdose prevention, competitive compensation and mental-health support systems (e.g., peer support lines and debriefing time). Investing in workforce wellbeing is essential not only for staff retention but also for the quality of client care; systemic harms of high caseloads and moral injury can undermine the person-centred- ethos of HF and contribute to service gaps.

An emphasis on peer support and the meaningful inclusion of PWLLE was a central theme of the workshop. This focus is supported by the literature, as peer workers have been shown to build mutual understanding and empathy through shared experience, a method that effectively motivates clients and reduces hierarchical barriers (32). However, peer workers often face precarious employment, heavy caseloads and role confusion, leading to high turnover (31). Our findings echoed these challenges and called for formal peer roles, fair compensation and ongoing training. Institutionalizing peer support within HF programs through stable positions, clear scopes of practice and supervisory support can enhance engagement, reduce stigma and ensure that programs remain accountable to the communities they serve.

### Limitations and future directions

This workshop provides valuable national insights into how best to support SUH needs within HF programs but has some limitations. Attendees were identified through a purposive expert-consultation approach based on the organizing committee's professional networks, prior collaborations, meetings, community and policy/practice connections, and relevant publications. Although 95 individuals representing 61 unique organizations were invited, 59 attended the workshop. This approach may have introduced selection bias, as individuals already connected to Housing First, SUH, harm reduction, or related policy and practice networks may have been more likely to be invited and to attend. In addition, individuals who declined, did not respond, or were otherwise disengaged from such initiatives may have held different perspectives from those represented in the workshop. As such, findings should be interpreted as expert consultation insights rather than representative views of all HF programs, stakeholders, or regional contexts across Canada. Furthermore, although the workshop was informed by review of the literature, the discussions primarily reflected self-reported practitioner and expert perceptions rather than objective, independent measures of program effectiveness. Future research should include longitudinal evaluations of implementation of integrated HF and SUH interventions, rigorous trials of SUH care integration into housing models and studies exploring funding and policy reforms. Comparative studies across regions and populations could clarify how context shapes challenges and opportunities, and how services can be tailored to meet unique, intersectional needs.

Although this workshop was not designed as a formal Participatory Action Research (PAR) study, our approach shared several PAR principles, including the active involvement of people with lived and living experience (PWLLE) as expert contributors, multi-stakeholder collaboration, and a focus on generating actionable knowledge to improve services. Future research should consider a formal PAR framework, which would involve PWLLE as co-researchers throughout the research process, from question formulation to dissemination, thereby strengthening rigor, ethical accountability, and the relevance of findings.

Relatedly, because detailed demographic data were not collected, we were unable to examine how the identified challenges and opportunities may differ across Indigenous, Black, racialized, 2SLGBTQ+, immigrant, or other minoritized groups. This is important because broader policy and service barriers related to homelessness and substance use health are shaped by structural racism, colonization, and their contemporary forms. Future research should examine these differential impacts using equity-oriented, anti-racist, decolonial, and participatory approaches.

## Conclusion

Integrating SUH care into HF programs is critical as harms from the unregulated supply, the substance use health and the housing crises converge. The roundtable's findings highlight that successful integration depends on person-centered, flexible, and well-resourced systems. Key priorities emerging from the discussion include overcoming siloed funding and services to create truly integrated care pathways that include the full spectrum of SUH care options to help people meet their individual goals; addressing the workforce crisis through better training, support, and compensation for staff; and meaningfully embedding peer support and lived expertise at all levels. While broader policy challenges, were noted as significant barriers, the immediate focus must be on building adaptable, collaborative, and adequately funded HF models. Without these core reforms, HF programs will continue to fall short for people with SUH support needs, perpetuating avoidable harms and preventable deaths.

## Data Availability

The original contributions presented in the study are included in the article/supplementary material, further inquiries can be directed to the corresponding author.
